# Chiral Mn^III^ (Salen) Covalently Bonded on Modified ZPS-PVPA and ZPS-IPPA as Efficient Catalysts for Enantioselective Epoxidation of Unfunctionalized Olefins

**DOI:** 10.3390/polym9030108

**Published:** 2017-03-17

**Authors:** Xiaochuan Zou, Cun Wang, Yue Wang, Kaiyun Shi, Zhongming Wang, Dongwei Li, Xiangkai Fu

**Affiliations:** 1 Department of Biological and Chemical Engineering, Chongqing University of Education, Nan’an 400067, China; wangcun5224@126.com (C.W.); b1083@163.com (Y.W.); shiky@cque.edu.cn (K.S.); wangzm@cque.edu.cn (Z.W.); dongweili001@gmail.com (D.L.); 2 The Key Laboratory of Applied Chemistry of Chongqing Municipality, College of Chemistry and Chemical Engineering, Southwest University, Beibei 400715, China; fxk@swu.edu.cn; 3 The Key Laboratory of Eco-Environments in Three Gorges Reservoir Region Ministry of Education, College of Chemistry and Chemical Engineering, Southwest University, Beibei 400715, China; 4 Research Institute of Applied Chemistry, College of Chemistry and Chemical Engineering, Southwest University, Beibei 400715, China

**Keywords:** Chiral Mn^III^ (salen) complex, hybrid zirconium phosphonate, covalent attachment, heterogeneous catalyst, asymmetric epoxidation

## Abstract

Chiral Mn^III^ (salen) complex supported on modified ZPS-PVPA (zirconium poly(styrene-phenylvinylphosphonate)) and ZPS-IPPA (zirconium poly(styrene-isopropenyl phosphonate)) were prepared using –CH_2_Cl as a reactive surface modifier by a covalent grafting method. The supported catalysts showed higher chiral induction (ee: 72%–83%) compared with the corresponding homogeneous catalyst (ee: 54%) for asymmetric epoxidation of *α*-methylstrene in the presence of 4-phenylpyridine *N*-oxide (PPNO) as axial base using NaClO as an oxidant. ZPS-PVPA-based catalyst 1, with a larger pore diameter and surface area, was found to be more active than ZPS-IPPA-based catalyst 2. In addition, bulkier alkene-like indene, was efficiently epoxidized with these supported catalysts (ee: 96%–99%), the results were much higher than those for the homogeneous system (ee: 65%). Moreover, the prepared catalysts were relatively stable and can be recycled at least eight times without significant loss of activity and enantioselectivity.

## 1. Introduction

The asymmetric epoxidation of alkenes into unique labile three-membered ether rings—which are useful organic building blocks for the synthesis of pharmaceuticals, agrochemicals, and fine chemicals—is one of the fundamental organic transformations [1,2,3]. Chiral Mn^III^ (salen) complexes, firstly reported by the Jacobsen [1,2] and Katsuki groups [4], have emerged as extremely efficient systems for the asymmetric epoxidation of unfunctionalized olefins. Although homogeneous catalysis is advantageous in terms of product yield and efficiency, it suffers from limitations of catalyst recovery and a problem with residual catalyst in the synthesized molecules. Thus, extension of these methods for large-scale synthesis—and for making pharmaceutically imperative molecules—becomes a matter of environmental and economic concern. Hence, it is of paramount importance that the utilization efficiency of these Mn^III^ (salen) complexes be improved by immobilizing them onto various heterogeneous supports [5,6,7,8,9,10,11,12,13,14] such as MCM-41, LDHS, silica, polymers, dendrimer, graphene oxide, glass beads, etc. The use of supported catalysts offers an attractive solution owing to their easy separation, convenient handing, non-toxic nature, and reusability.

Our group has, in recent decades, been concerned with the research of organic polymer-inorganic phosphate salt (Zr, Zn, Al, and Ca) hybrid materials [15,16,17,18,19,20]. The features of these organic polymer-inorganic phosphate salts (Zr, Zn, Al, and Ca) supports are different from either common polystyrene or pure phosphate salts (Zr, Zn, Al, and Ca). They consist of polystyrene which is easily modified and hydrophobic, phosphate salt (Zr, Zn, Al, and Ca) parts that are hydrophilic, and their nanometer-scale self-assembled layered structure. Thus, a great number of polystyrene segments combined with layered phosphate salts (Zr, Zn, Al, and Ca) will lead to the formation of different caves, holes, pores, micropores, channels, and secondary channels with various sizes and shapes which presents an opportunities for support material candidates.

**Scheme 1:**
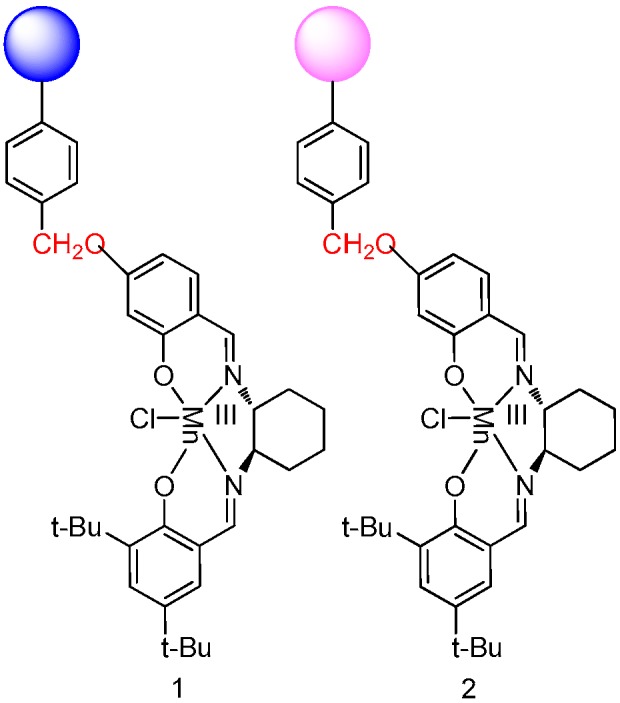
Illustration of the ZPS-PVPA-based catalyst 1and ZPS-IPPA-based catalyst 2.

Recently [21,22], we have devoted ourselves to axially immobilizing Jacobsen’s catalyst on a series of organic polymer—inorganic phosphate salts (Zr, Zn, Al, and Ca) through different diamine, polyamine, diol, or diphenoxyl linkers, the prepared heterogeneous catalysts were evaluated for enantioselective epoxidation of unfunctionalized olefins. Representative works [17] indicated that ZPS-PVPA-based catalyst-effectively catalyzed epoxidation of styrene and *α*-methylstyrene (ee: 50% to 78% and 86% to >99%) with *m*-CPBA or NaClO. These results are significantly better than those achieved with the homogeneous chiral catalysts under the same reaction conditions (ee: 47% and 65%). Moreover, the immobilized catalysts could be reused at least 10 times without significant loss of activity and enantioselectivity. Furthermore, a point worth emphasizing is that ZPS-PVPA-based catalyst results in remarkable increase of conversion and ee values in the absence of expensive O-coordinating axial additive for the asymmetric epoxidation of olefins [23,24], which is exactly opposite to the literature reported earlier for both homogeneous and heterogeneous systems [25,26]. This novel additive effect was mainly attributed to the support ZPS-PVPA and the axial phenoxyl linker group. Further research demonstrated that similar catalytic results were dependent on the axial phenoxyl or oxyalkyl linker group but also other organic polymer-inorganic hybrid phosphate salts (Zn, Al, and Ca). In order to better understand this novel additive effect and for the sake of searching for different stable, efficient, and reusable heterogenous Mn^III^ (salen) catalysts we are encouraged to conduct further research. Herein, we describe the covalent bonding of an asymmetric chiral Mn^III^ (salen) complex 3 which was rarely reported in our group on modified hybrid zirconium phosphonate ZPS-PVPA and ZPS-IPPA to give supported complexes 1 and 2 (Scheme 1). To allow the maximum conformational mobility in the complex needed to obtain a high level of asymmetric induction and prevent leaching of active complex, the grafting was done through one side of the 4-position in chiral salen ligand. The enantioselective catalytic activities of the catalyst 1 and 2 were examined for epoxidation of *α*-methylstyrene, styrene, and indene in the presence/absence of axial base. Depressingly, the good catalytic efficiency is with the help of an expensive axial base in different oxidant systems. In turn, the results further indicate that the novel additive effects were attributed to the axial phenoxyl or oxyalkyl linker groups but not supports and the covalent attachment method.

## 2. Material and Methods

### 2.1. Materials

(1R,2R)-(-)-1,2-Diaminocyclohexane, *α*-methylstyrene, indene, *n*-nonane, 4-phenylpyridine *N*-oxide (PPNO), *N*-methylmorpholine *N*-oxide (NMO), and *m*-chloroperbenzoic acid (*m*-CPBA) were supplied by Alfa Aesar (Tianjin, China). Other commercially available chemicals were laboratory-grade reagents from local suppliers. Styrene was passed through a pad of neutral alumina before use. Jacobsen′s catalyst 4 was synthesized according to the standard literature procedures [1].

### 2.2. Methods

FT-IR spectra were recorded from KBr pellets using a Bruker RFS100/S spectrophotometer (Bruker, Germany) and diffuse reflectance UV-vis spectra of the solid samples were recorded in the spectrophotometer (Bruker, Germany) with an integrating sphere using BaSO_4_ as standard. X-ray photoelectron spectrum was recorded on ESCALab250 instrument (Thermo Fisher, Waltham, MA, USA). Elemental analysis was measured by PE-2400 (C.H.N.) (PerkinElmer, Waltham, MA, USA). TG analyses were performed on an SBTQ600 Thermal Analyzer (LINSEIS, Robbinsville, NJ, USA) with the heating rate of 20 °C·min^−1^ from 25 to 1000 °C under flowing N_2_ (100 mL·min^−1^). The Mn contents of the catalysts were determined by a TAS-986G atomic absorption spectroscopy (Pgeneral, Beijing, China). SEM were performed on KYKY-EM3200 microscopy (KYKY, Beijing, China). TEM were obtained on a TECNAI10 apparatus (PHILIPS, Amsterdam, Holland). Nitrogen adsorption isotherms were measured at 77 K with a 3H-2000I volumetric adsorption analyzer (Huihaihong, Beijing, China) using BET method. The racemic epoxides were prepared by epoxidation of the corresponding olefins by 3-chloroperbenzoic acid (*m*-CPBA) in CH_2_Cl_2_ and confirmed by NMR (BrukerAV-300, Bruker, Germany), and the gas chromatography (GC) was calibrated with the samples of *n*-nonane, olefins, and corresponding racemic epoxides. The yields (with *n*-nonane as internal standard) and the ee values were analyzed by gas chromatography (GC) with a Shimadzu GC2014 instrument (Shimadzu, Kyoto, Japan) equipped using a chiral column (HP19091G-B233, 30 m × 0.25 mm × 0.25 μm) and FID detector, injector 230 °C, detector 230 °C. The column temperature for *a*-methylstyrene, styrene, indene was 80–180 °C. The retention times of the corresponding chiral epoxides are as follows: (a) *α*-methylstyrene epoxide: the column temperture is 80 °C, *t*_S_ = 12.9 min, *t*_R_ = 13.0 min; (b) styrene epoxide: the column temperture is 80 °C, *t*_R_ = 14.7 min, *t*_S_ = 14.9 min; (c) indene epoxide: the column temperature is programmed from 80 to 180 °C, *t*_SR_ = 16.1 min, *t*_RS_ = 17.1 min.

## 3. Preparation of Catalysts

### 3.1. Synthesis of ZCMPS-PVPA and ZCMPS-IPPA

**Scheme 2:**
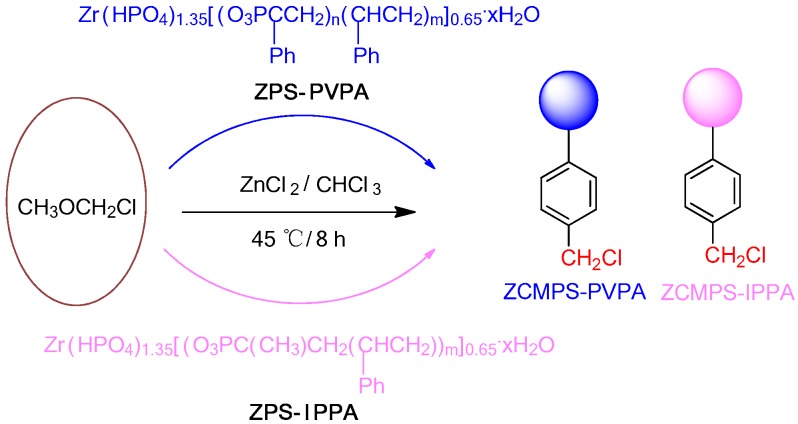
Synthesis of ZCMPS-PVPA and ZCMPS-IPPA.

The synthesis and characterization of chloromethylated-zirconium poly(styrene-phenylvinylphosphonate)-(ZCMPS-PVPA) and chloromethyl-zirconium poly(styrene-isopropenyl phosphonate)-(ZCMPS-IPPA) have been reported upon earlier by our group [16,17] (Scheme 2).

Chloromethyl methyl ether (8.22/12.5 g), anhydrous zinc chloride (1.6/1.25 g) and ZSP-PVPA/ZPS-IPPA (5.0 g) were mixed and stirred at 45 °C for 8 h. After cooling, a small amount of water and methanol was added into the mixture, filtered, washed with methanol and acetone, and dried in vacuo to obtain ZCMPS-PVPA, yield: 90.3%, IR (KBr cm^−1^): 3026, 2925 (CH), 2337 (O=P–OH), 1605, 1545, 1512, 1495 (–C_6_H_5_), 1271 (P=O), 706 (C–Cl) cm^−1^, ZCMPS-IPPA, yield: 92.3%, IR (KBr cm^−1^): 3028, 2920 (CH), 2328 (O=P–OH), 1602, 1540, 1511, 1490 (–C_6_H_5_), 1268 (P=O), 705 (C–Cl) cm^−1^.

### 3.2. Synthesis of Asymmetric Chiral Salen Ligand

A 100 mL flask was charged with (1R,2R)-1,2-diaminocyclohexane monohydrochloride salt (151 mg, 1.0 mmol), activated 4 Å molecular sieves (200 mg), and anhydrous methanol/dichloromethane (1:1, 10 mL). 2,4-dihydroxybenzaldehyde (138 mg, 1.0 mmol) was added in one portion, the reaction mixture was stirred at room temperature for 4 h. A solution of 3,5-Di-tertbutyl-2-hydroxy-benzaldehyde (234 mg, 1.0 mmol) resolved in methanol/dichloromethane (1:1, 10 mL) was added to the reaction mixture, followed by the slow addition of triethylamine (0.27 mL, 2.0 mmol). The reaction mixture was stirred for an additional 4 h followed by the removal of the solvents. The residue was dissolved in dichloromethane (20 mL), washed with water (2 × 20 mL), and dried with magnesium sulfate. Flash chromatography of the crude product on silica gel (ether/hexanes = 1:4 to 1:1) afforded asymmetric chiral salen ligand (0.27 g, Yield: 60%) as a light yellow solid [27]. ^1^H NMR (300 MHz, CDCl_3_) δ = 1.32 (s, 9H), 1.40 (s, 9H), 1.68 (m, 4H), 1.89 (m, 4H), 3.29 (m, 2H), 6.33 (m, 1H), 6.63 (m, 1H), 6.87 (m, 1H), 6.98 (d, 1H), 7.34 (d, 1H), 8.22 (s, 1H), 8.29 (s, 1H).

### 3.3. Synthesis of Asymmetric Chiral Mn^III^ Salen Complex 3 

**Scheme 3:**
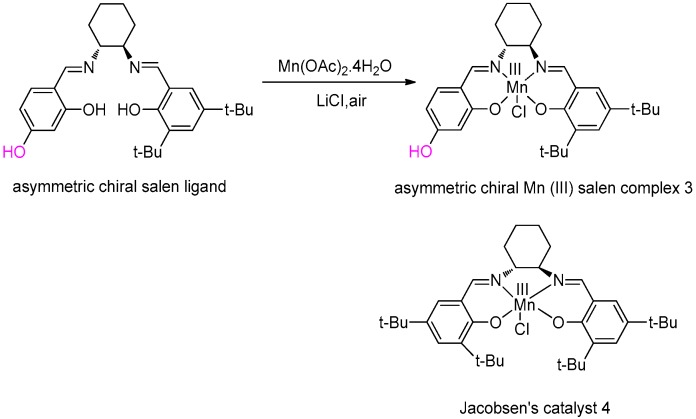
Illustration of asymmetric chiral Mn^III^ (salen) complex 3 and Jacobsen’s catalyst 4.

Manganese inserted into the salen ligand was accomplished by adding a solution of Mn (OAc)_2_·4H_2_O (103.0 mg, 0.42 mmol) in 10 mL of ethanol to the salen ligand 2 (94.9 mg, 0.21 mmol) with stirring (Scheme 3). The mixture was refluxed for 3 h under the protection of Ar. Then air was bubbled for an additional 2 h, and 26.7 mg of solid LiCl was added. After refluxing for 1 h, the bead was filtered and rinsed sequentially with CH_2_Cl_2_, ethanol, and H_2_O, and then finally dried in vacuum to yield brown power 3 [27], Yield: 90.0%, mp: 318 °C, IR (KBr cm^−1^): 1624 (C=N), Anal. Calcd for complex 3 (C_28_H_36_MnClN_2_O_2_): C, 70.2%; H, 6.45%; N, 5.01%. Found: C, 74.15%; H, 8.42%; N, 4.17%. Jacobsen’s catalyst 4, Yield: 90.26%, m.p. 331.4 °C, IR (KBr cm^−1^): 1622 (C=N). Anal. Calcd for complex 4(C_36_H_52_MnClN_2_O_2_): C, 67.05%; H, 8.31%; N, 4.12%. Found: C, 69.03; H, 8.65%; N, 3.99%.

### 3.4. Immobilization Asymmetric Chiral Mn^III^ (Salen) Complex 3 on the ZCMPS-PVPA and ZCMPS-IVPA

**Scheme 4:**
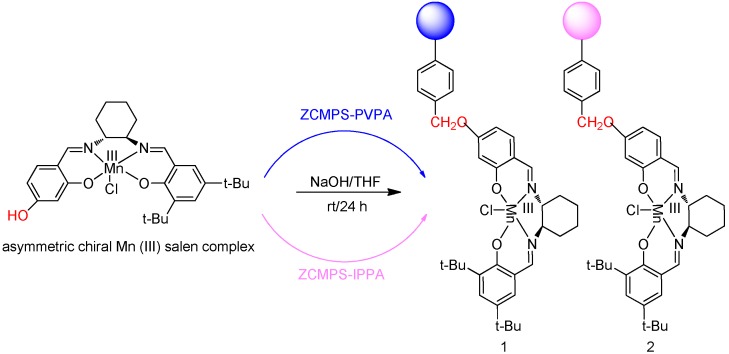
Synthesis of the immobilized catalysts.

Catalyst 3 (543 mg, 1.0 mmol) and NaOH (120 mg, 3 mmol) were added to a suspension of ZCMPS-PVPA and ZCMPS-IVPA (0.50 mmol Cl) that had been pre-swelled in 10 mL of dry THF for 30 min (Scheme 4). The yellow suspension was refluxed for 24 h. The dark brown powder was collected by filtration and washed thoroughly with ethanol, CH_2_Cl_2_ and deionized water respectively and dried in vacuo. The CH_2_Cl_2_ filtrate was detected by UV-vis until no peaks could be detected (with pure CH_2_Cl_2_ solvent as reference). Yield: 88.0%, 86.1% respectively. The loading of Mn^III^ (salen) complex 3 in the heterogenized catalyst, based on the Mn element, was 0.43–0.52 mmol/g determined by AAS.

Catalyst 1: IR (KBr cm^−1^): 2925–2880 (C–H), 1620, 1495, 1450 (–C_6_H_5_), 1623–1626 (C=N): DR UV-Vis (nm): 250, 424. AAS (mmol/g): 0.52;

Catalyst 2: IR (KBr cm^−1^): 2945–2880 (C–H), 1625, 1500, 1455 (–C_6_H_5_), 1624–1636 (C=N). DR UV-Vis (nm): 253, 425.5. AAS (mmol/g): 0.43.

### 3.5. Asymmetric Epoxidation

Enantioselective epoxidation reactions were carried out using catalysts 1–2 (0.025 mmol)with *α*-methylstyrene, styrene, and indene (0.5 mmol) as substrates in 3 mL of dichloromethane under reaction conditions in the presence of PPNO (0.19 mmol) as an axial base with aqueous buffered 1.8 mL NaClO (0.55 mol/L pH = 11.3) as an oxidant, the aqueous buffer was prepared with commercially available sodium hypochlorite (10%) diluted by 0.05 mol/L sodium dihydrogen phosphate (V_NaClO_:V_NaH2PO4_ = 25:10), then, the solution was adjusted by 1.00 mol/L hydrochloric acid to pH = 11.5. When the conversion was steady, the mixture was diluted with CH_2_Cl_2_ (3 mL). The phases were separated and the aqueous layer was extracted with CH_2_Cl_2_ (3 mL × 2). The combined organic layer was washed with brine (3 mL × 2) and dried over anhydrous sodium sulfate. The concentrated filtrate was purified by chromatography on a silica gel column to afford the corresponding epoxide. For *m*-CPBA/NMO system, a solution of alkene (0.5 mmol), NMO (337.5 mg, 2.5 mmol), *n*-nonane (internal standard, 90.1 mL, 0.5 mmol), and immobilized Mn^III^ (salen) complexes (0.025 mmol, 2.0 mol %) in CH_2_Cl_2_ (3 mL) was cooled to the desired temperature. Solid *m*-CPBA (172.5 mg, 1.0 mmol) was added in four portions over 2 min. After completion of the reaction, the mixture was washed sequentially with saturated sodium hydroxide and brine—to remove any residual *m*-CPBA and the corresponding acid—and dried over anhydrous Na_2_SO_4_. The conversion and ee values were determined by GC using nonane as an internal standard.

## 4. Results and Discussion

### 4.1. FT-IR Spectroscopy

**Figure 1:**
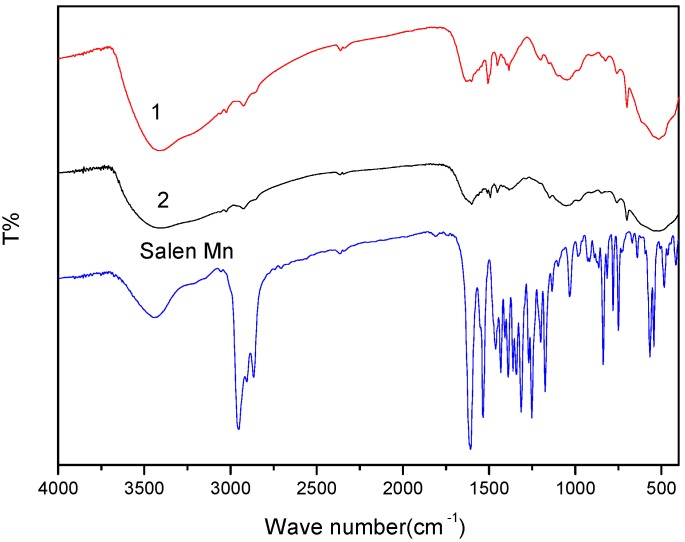
FT-IR spectra of catalysts 1 and 2 and chiral Mn^III^ (Salen) (Jacobsen’s catalyst 4).

FT-IR spectra (Figure 1) of the supported catalysts 1 and 2 showed bands at near 1620 cm^−1^ due to the C=N stretching vibration which was similar with homogeneous chiral Mn^III^ (salen) catalysts. Besides, the IR band of the supported catalysts at near 1026 and 1473 cm^−1^ is attributed to Ph–O–C stretching vibrations which appeared in the support of ZCMPS-PVPA and ZCMPS-IPPA. This results preliminary showed that the chiral Mn^III^ (salen) molecules are immobilized on these two kinds of carriers.

### 4.2. DR UV-Vis Spectroscopy

**Figure 2:**
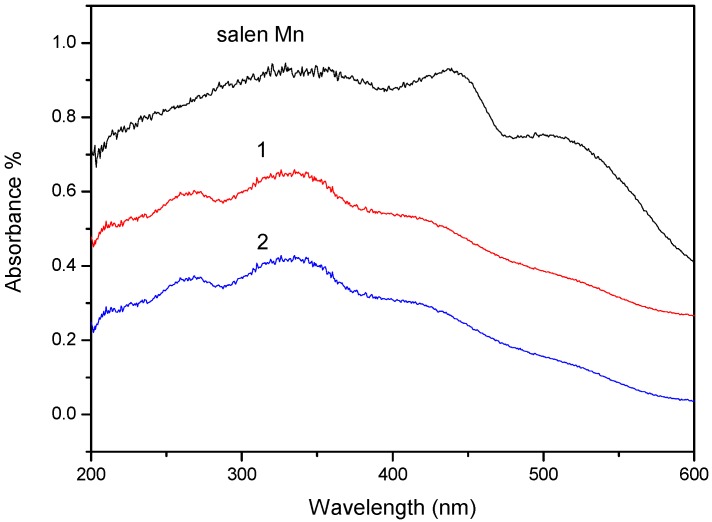
UV-vis spectra of catalyst 1 and 2 and chiral Mn^III^ (Salen) (Jacobsen’s catalyst 4).

The UV-vis spectra (Figure 2) of the supported catalysts 1 and 2 showed characteristic bands in homogeneous Mn^III^ (Salen), indicating the presence of Mn^III^ (Salen) in ZCMPS-PVPA and ZCMPS-IPPA. The characteristic bands for Mn^III^ (Salen) at 334, 435 and 510 nm had been blue-shifted to near 330, 410 and 505 nm after immobilizing, respectively. The blue-shifting was mainly due to the interaction between the carrier and the chiral Mn^III^ (salen). Hence, the diffuse reflectance UV-vis spectra also gave further evidence for successful immobilization.

### 4.3. Microscopic Analysis

**Figure 3:**
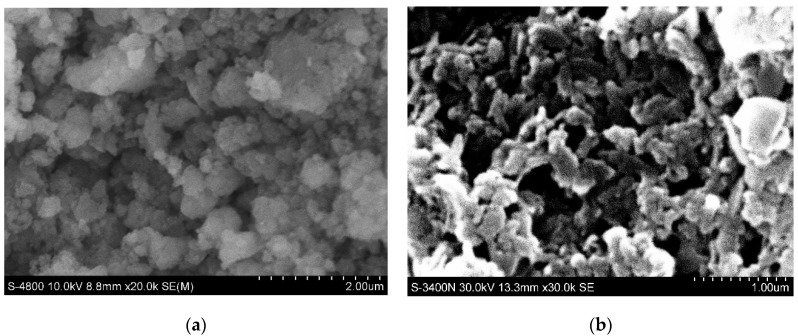
SEM photograph of (**a**) catalyst 1; (**b**) catalyst 1 (treated in basic solution).

**Figure 4:**
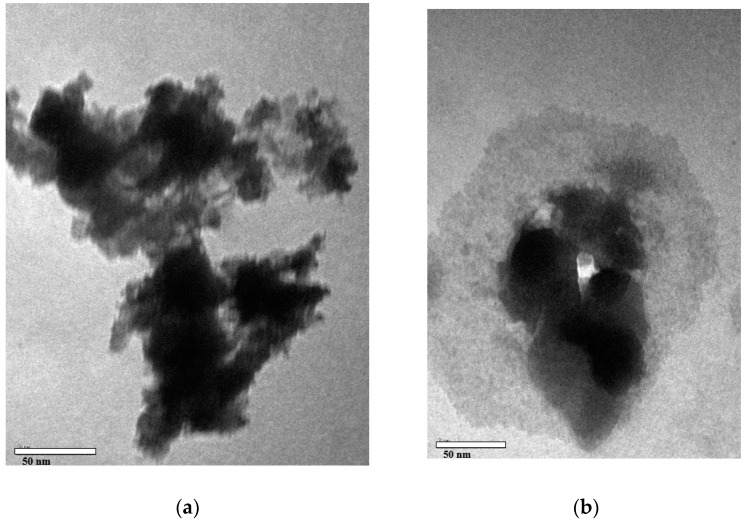
TEM photograph of (**a**) catalyst 1; (**b**) catalyst 1 (treated in basic solution).

Scanning electron microscopy (SEM) of catalyst 1 (Figure 3a,b) shows the surface morphology of the catalyst, in which Figure 3a indicates that the amorphous catalyst with particle diameter of one hundred to several hundred nanometers, and each particle is consisted of several smaller particles with diameters of dozens of nanometers. These smaller particles with different shapes gather together irregularly, some micopores, cavums, and secondary channels which increase the surface area of the catalyst and provide enough space accessibly for substrates oxidant molecules in the catalytic active sites are clearer in Figure 3a. However, the catalyst treated in basic solution (Figure 3b) is relatively looser than that depicted in Figure 3a. From the SEM in Figure 3b, the particles of the base treated catalyst with diameter dozens nanometers are smaller, and the micropores, cavums and secondary channels are larger than that of in Figure 3a. Transmission electron microscopy (TEM) exhibits that the average diameter of these secondary channels among the layers of the catalyst is around 50 nm (Figure 4a,b). It is deduced that the inorganic Zirconium (ZrHPO_4_) parts of the catalyst that show layered structure at a nanoscale were enlarged and decomposed in basic solution (Figure 4b), and the special configurations of the catalysts could be beneficial to the substrates approaching the internal catalytic active sites easily and offer enough space for the epoxidation of olefins.

### 4.4. The BET Surface Areas, Pore Volumes, and Average Pore Sizes of ZCMPS-PVPA, ZCMPS-IPPA, and 1

Date on BET surface area, average pore size, and pore volume are presented in Table 1. A large decrease in BET surface area was observed in catalysts 1 and 2 prepared by covalent of the chiral salen ligand at one side of 4-position with the –CH_2_Cl of hybrid zirconium phosphonate-(ZPS-PVPA and ZPS-IPPA), with a reduction in the pore volumes and average pore sizes, suggesting that the Mn^III^ (salen) was mainly located on the inner channels of support material. Moreover, the surface area (ZCMPS-PVPA vs. ZCMPS-IPPA; 120.3 vs. 100.3 m^2^/g) provide the substrates with enough opportunity to approach the catalytic active sites.

### 4.5. X-ray Photoelectron Spectroscopy

**Figure 5:**
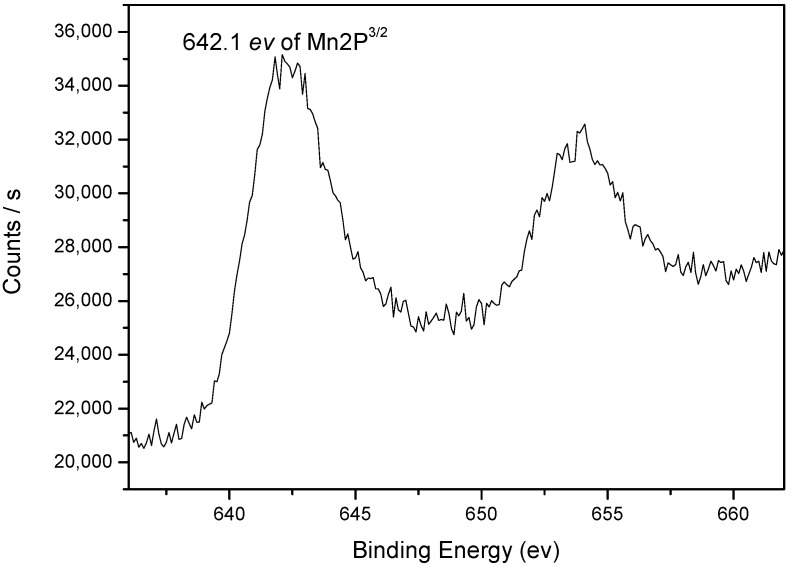
XPS spectra of catalyst 1.

XPS spectrum (Figure 5) gives further evidence for successful immobilization based on the fact that the characteristic bond at 642.1 eV of Mn2P^3/2^ is clear, which is in agreement with the values previously reported for Mn^III^ (Salen) and porphyinic ligands [28,29].

### 4.6. The Activities of the Catalysts

**Scheme 5:**
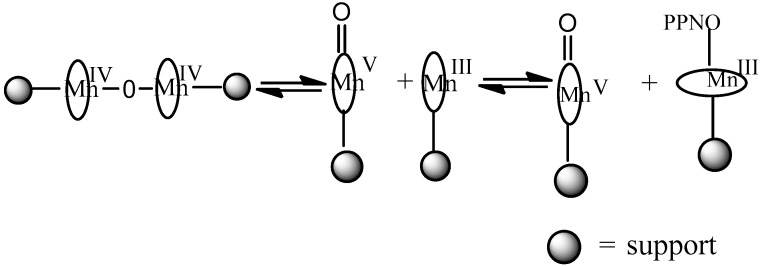
The different valence states of Mn=O*^n^*^+^ in the forming of active intermediates.

The enantioselective catalytic activities of the chiral Mn^III^ (salen) complex and catalysts 1 and 2 were examined for epoxidation of *α*-methylstyrene, styrene, and indene, at 0 °C using aqueous NaOCl as an oxidant in the presence/absence of PPNO as an axial base. The data reported in Table 2 indicate that all reactions proceeded smoothly, and the ZPS-PVPA-based catalyst 1—with a larger pore diameter and surface area—was found to be more active than ZPS-IPPA-based catalyst 2 (ee: 83% vs. 72%, con: 90% vs. 75%). Significantly, the enantio-induction was high with α-methylstyrene, styrene and indene in this study, which were higher ee values than the homogeneous catalyst under the same conditions. The increase in chiral recognition could arise from the unique spatial environment created by both the chiral salen complex and the surface of the supports used. Similar results were obtained by Kim [30] and Li [6], respectively. Kim et al. reported that, for the asymmetric epoxidation of α-methylstyrene, the ee increased from 51% to 59% after immobilization of Mn^III^ (salen) on the siliceous MCM-41 by multi-step grafting. Hutchings [31] found that the confinement effect originated from the zeolite cage could improve the chiral induction for the asymmetric epoxidition of styrene. It was deduced that the data of increase in enantiomeric excess is mainly attributed to the microenvironment effects of ZPS-PVPA and ZPS-IPPA immobilized Mn^III^ (salen) [16,17,22,23], which result from the layered structure, micoropores, and channels; the hydrophilic property of polystyrenylphosphonate parts; and hydrophobic of zirconium parts of the hybrid zirconium phosphonate. These features are different from either pure polystyrene or pure zirconium. In the absence of PPNO, the catalyst 1 oxidize *α*-methylstyrene (ee*:* 33%, con: 25%), suggests that the presence of PPNO is essential for catalyst stability and enantioselectivity. PPNO, which is only weakly bound to the manganese center, has remarkable effects on both the activity and enantioselectivity of the enantioselective epoxidation by activating and stabilizing the catalyst. We think there are two possibilities as to why PPNO can coordinate to manganese [16,17]. The first we propose is that the effect of PPNO is due to a set of equilibria (Scheme 5), wherein the active (salen) Mn^V^=O complex undergoes reversible coupling with a Mn^III^ complex to generate an inactive *u*-oxo dimer. In the presence of PPNO, the equilibrium is shifted toward the Mn^V^ oxo intermediate as a result of additive binding to the coordinatively unsaturated Mn^III^ complex. Acceleration in the rate of epoxidation is then expected due to the increased concentration of the active Mn^V^ oxo in solution. Another reason is that we think that the PPNO does not really coordinate with Mn, being only weakly bound to the manganese center, and that this has remarkable effects on enantioselectivity of the enantioselective epoxidation by increasing in stability of the Mn^V^ oxo intermediate.

The role of PPNO as an axial base was established earlier by others authors [25,26,32,33]. ZCMPS-PVPA and ZCMPS-IPPA alone showed negligible catalytic activity toward epoxidation of *α*-methylstyrene taken as a representative substrate.

Furthermore, catalysts 1 and 2 were also found to be efficient in the epoxidation of indene (ee*:* 96%–99%), with results higher than those from the homogeneous reaction carried out with catalyst 4 under identical reaction conditions (ee: 65%). However, the catalytic reactions were found to be slower (12 h) in catalysts 1 and 2, similar results were observed in epoxidation of *α*-methylstyrene and styrene. This behavior was attributed to the diffusional constraints usually present when a catalyst is supported inside the materials. Materials with larger pore sizes would be expected to face less diffusional resistance. Thus, the higher TOF values obtained with catalyst 1 supported on ZPS-PVPA compared with catalyst 2 supported on ZPS-IPPA are to be expected.

**Figure 6:**
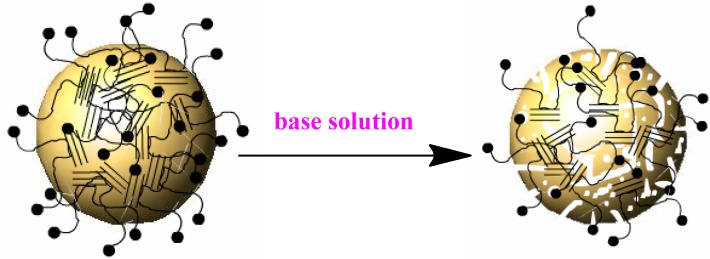
The ideal decomposed process of catalyst 1. 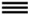 Zirconium phosphates (phosphonates); 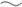 Chloromethyl poly (styrene-phenylvinylphosphonate); ● Mn^III^ (salen).

The observed conversions and ee values of epoxides are well with catalyst 1 and 2 using NaClO as oxidant. When α-methylstyrene was used as substrate, the ee values of the resulting epoxides were found to be 72%–83%, with conversions of 75%–90%, while only 43% ee values was obtained using *m*-CPBA as oxidant (Table 3). A possible explanation for this phenomena may be the layers of ZPS-PVPA or ZPS-IPPA are enlarged or even decomposed in base solution (the PH value of NaClO oxidation system is 11.35). The decomposed process of catalyst 1 in the base solution condition was deduced and shown in Figure 6, which was indicated that one amorphous particle of the immobilized catalyst 1 are consisted irregularly by hundreds or thousands of smaller particles of catalyst microcrystaline with regular layers, and the most of the catalytic sites immobilized and embed on the surface, in the interlayer or interlamellar region, or among the microcrystaline of ZPS-PVPA under adequate conditions. While in the base solution, the layers of ZPS-PVPA are expanded or even partly decomposed and more secondary channels are formed, and the original secondary channels are enlarged, so some of the embed catalytic active sites were exposed in the base reaction solution, the substrates and the reactants could diffuse to these catalytic sites easily through these secondary channels. However, in the *m*-CPBA oxidation system which is not in base reaction solution, the nano-particles of immobilized catalyst gather together, and the structure of the catalyst is rigid and very stable with relatively fewer secondary channels, and some of the embedded catalytic active sites cannot work effectively.

### 4.7. The Reusability of the Catalyst

The recyclability of catalyst 1 was carried for the epoxidation of *α*-methylstyrene as a representative substrate. After the first run of the epoxidation reaction, catalyst 1 was separated by centrifugation. The separated catalyst was washed thoroughly with dichloromethane, dried, and subjected to another cycle with fresh reactants under similar epoxidation conditions. Table 4 shows the results of the recovery and reusability of catalyst 1. To our delight, catalyst 1 could be reused eight times with no appreciable decrease in yield and enantioselectivity of *α*-methylstyrene epoxide. Chemical analysis of the Mn content in the supernatant revealed no detectable leaching of Mn species during the reaction. The results suggested excellent stability and reusability of catalyst 1 under the basic reaction conditions in this work.

## 5. Conclusions

Catalysts 1 and 2 were prepared by heterogenizing asymmetric chiral Mn^III^ (salen) complex onto modified hybrid materials ZPS-PVPA and ZPS-IPPA by using a covalent bonding method. The supported catalysts 1 and 2 effectively catalyzed epoxidation of *α*-methylstrene (ee: 72%–83%) with aqueous NaClO in the presence of 4-phenylpyridine *N*-oxide (PPNO) as axial base. These results are significantly better than those achieved with the catalyst 4 under a homogeneous system (ee: 54%). Remarkably, catalysts 1 and 2 worked well for relatively bulkier alkene such as indene (ee: 96%–99%), and the results were much higher than those for the homogeneous system (ee: 65%). Moreover, the prepared catalysts are relatively stable and can be recycled at least eight times without significant loss of activity and enantioselectivity. Depressingly, the good catalytic efficiency is with the help of expensive O-coordinating axial additive in different oxidant systems. In turn, the results further indicated that the novel additive effects were attributed to the axial phenoxyl or oxyalkyl linker groups but not supports and the covalent attachment method.

## Figures and Tables

**Table 1:** The BET surface areas, pore volumes, and average pore sizes of ZCMPS-PVPA, ZCMPS-IPPA, and 1 ^[a]^.

Sample	Surface area (m^2^/g) ^[b]^	Pore volume (×10^−1^ cm^3^/g) ^[c]^	Average pore size (nm) ^[d]^
ZCMPS-PVPA	120.3	0.27	38.1
1	86.0	0.18	27.3
ZCMPS-IPPA	100.3	0.22	27.9
2	68.2	0.15	23.6

^[a]^ The samples were out gassed at 150 °C for 6 h; ^[b]^ Surface area based on multipoint BET method; ^[c]^ Pore volume based on the desorption data of BJH method; ^[d]^ Pore size based on the desorption data using BJH method.

**Table 2:** Asymmetric epoxidation of different substrates by homogeneous and heterogeneous catalysts with NaClO as oxidant systems ^a^.

Entry	Substrate	Catalyst	t/h	Conversion (Selectivity) ^b^ (%)	ee (%) ^c^	TOF ^d^ × 10^−4^ (s^−1^)
1	α-methylstyrene	Jacobsen’s catalyst	6	99 (99)	54 ^f^	11.3
2		1	12	90 (80)	83 ^f^	9.38
3		2	12	75 (85)	72 ^f^	7.38
4		1 ^e^	12	25 (29)	33 ^f^	0.84
5		ZCMPS-PVPA	24	8	-	-
6		ZCMPS-IVPA	24	6	-	-
7	styrene	Jacobsen’s catalyst	6	>99 (99)	34.5 ^g^	22.7
8		1	12	>99 (88)	58.5 ^g^	10.1
9		2	12	88 (73)	42.8 ^g^	7.43
10	indene	Jacobsen’s catalyst	6	98 (>99)	65 ^h^	22.46
11		1	12	65 (80)	99 ^h^	6.02
12		2	12	52 (68)	96 ^h^	4.09

^a^ Reactions were performed in CH_2_Cl_2_ (4 mL) with substrates (1.0 mmol), *n*-nonane (1.0 mmol), catalysts 1 and 2, and homogeneous Mn^III^ (salen) (based on Mn, 0.02 mmol), NaClO (pH = 11.3, 4-PPNO: 0.38 mmol); ^b^ Conversions were determined by GC, by integration of product peaks against an internal quantitative standard, correcting for response factors; ^c^ Determined by GC with a chiral capillary column HP 19091G-B233; ^d^ Turnover frequency (TOF) is calculated by the expression of [product]/[catalyst] × time (s^−1^); ^e^ Reaction conducted in absence of PPNO; ^f^ Expoxide configuration *S*; ^g^ Expoxide configuration *S*; ^h^ Expoxide configuration 1*S*, 2*R*.

**Table 3:** Asymmetric epoxidation of *α*-methylstyrene by catalyst 1 with *m*-CPBA as oxidant systems ^a^.

Entry	t/h	Conversion (selectivity) ^b^ (%)	ee (%) ^c^	TOF ^d^ × 10^−4^ (s^−1^)
1	1	60(45)	55 ^f^	37.5
2	1 ^e^	26(20)	18 ^f^	7.22
3	2	66(47)	54 ^f^	21.54
4	12	70(41)	43 ^f^	3.32

^a^ Reactions were carried out in CH_2_Cl_2_ (3.0 mL) with *m*-CPBA (1.0 mmol) and NMO (2.5 mmol, if necessary), nonane (internal standard, 0.5 mmol), and catalyst 1 (based on Mn, 0.02 mmol); ^b^ Conversions were determined by GC, by integration of product peaks against an internal quantitative standard, correcting for response factors; ^c^ Determined by GC with a chiral capillary column HP 19091G-B233; ^d^ Turnover frequency (TOF) is calculated by the expression of [product]/[catalyst] × time (s^−1^); ^e^ Reaction conducted in absence of NMO; ^f^ Expoxide configuration *S*.

**Table 4:** Recycling data for enantioselectivity epoxidation of *α*-methylstyrene as representative substrate using catalyst 1 ^a^.

Run	t (h)	Conversion (selectivity) ^b^ (%)	ee (%) ^c^	TOF ^d^ × 10^−4^ (s^−1^)
1	12	90 (80)	83 ^e^	9.38
2	12	89 (80)	81 ^e^	8.24
3	12	86 (81)	81 ^e^	8.06
4	12	85 (79)	79 ^e^	7.77
5	12	79 (76)	78 ^e^	6.95
6	12	76 (77)	77 ^e^	6.77
7	12	77 (72)	76 ^e^	6.42
8	12	76 (65)	72 ^e^	5.72
9	12	71 (61)	63 ^e^	5.01
10	12	63 (54)	58 ^e^	3.94
11	24	62 (49)	53 ^e^	3.52

^a^ Reactions were performed in CH_2_Cl_2_ (4 mL) with substrates (1.0 mmol), *n*-nonane (1.0 mmol), catalysts 1 and 2, and homogeneous Mn^III^ (salen) (based on Mn, 0.02 mmol), NaClO (pH = 11.3, 4-PPNO: 0.38 mmol); ^b^ Conversions were determined by GC, by integration of product peaks against an internal quantitative standard, correcting for response factors; ^c^ Determined by GC with a chiral capillary column HP 19091G-B233; ^d^ Turnover frequency (TOF) is calculated by the expression of [product]/[catalyst] × time (s^−1^); ^e^ Expoxide configuration *S*.
